# Diverse assays from a single skin punch biopsy to assess topical drug intervention

**DOI:** 10.1111/bjd.17353

**Published:** 2019-01-20

**Authors:** M. Danilenko, K. Hodgson, R. Stones, A. Husain, M. Zangarini, G. Veal, N. Rajan

**Affiliations:** ^1^ Institute of Genetic Medicine Newcastle University Newcastle upon Tyne U.K.; ^2^ Department of Pathology Royal Victoria Infirmary Newcastle upon Tyne U.K.; ^3^ Northern Institute for Cancer Research Newcastle University Newcastle upon Tyne U.K.; ^4^ Department of Dermatology Royal Victoria Infirmary Newcastle upon Tyne U.K.

Dear Editor, The development of targeted topical treatments for inherited skin tumour syndromes, such as naevoid basal cell carcinoma syndrome, is desirable because this approach avoids surgery and is unlikely to cause systemic side‐effects.^1^ However, in CYLD cutaneous syndrome (CCS) (also known as Brooke–Spiegler syndrome), transgenic mouse models fail to recapitulate the human phenotype,^2^ hampering similar translational advances for these patients. Research methodologies that offer drug penetration data in addition to evidence of ‘on target’ drug effects in topically treated human CCS tumour samples are informative in the absence of relevant mouse models. Here we describe such a method, employing serial sections of human skin tumour punch biopsies, which is relevant to the study of topical interventions in CCS and can be used in other skin diseases.

**Figure 1:**
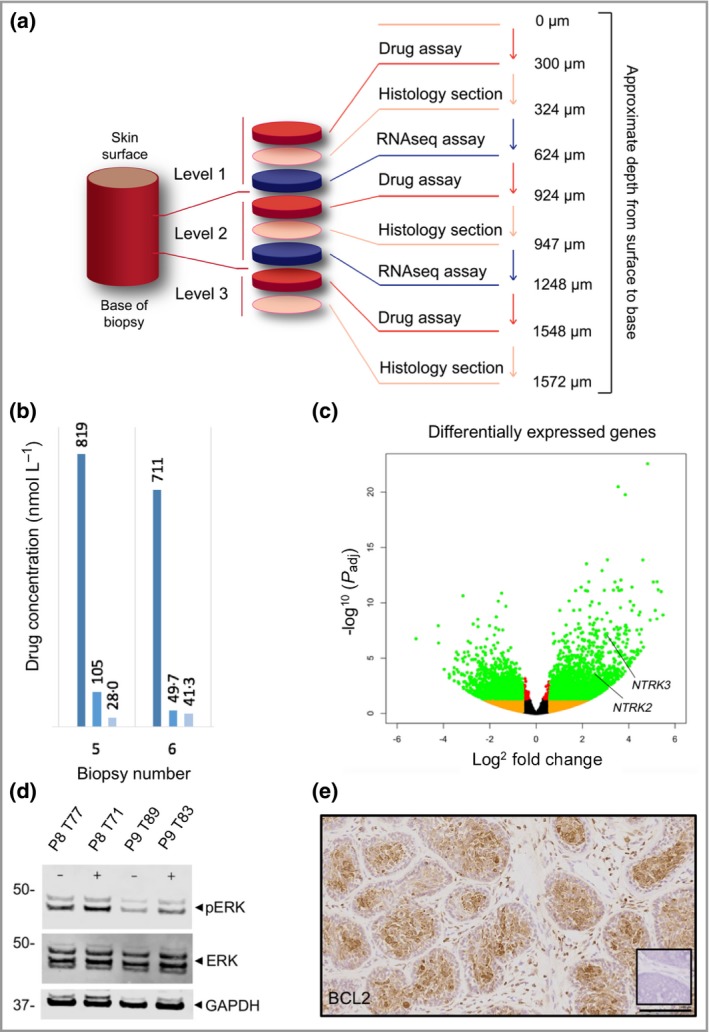
Diverse and complementary assays from a single skin biopsy. (a) Diagram indicating the serial cryosectioning sequence used and the levels studied. (b) Drug concentrations quantified in tissue sections at three levels in samples from two patients (level 1, dark blue bar; level 2, medium blue bar; level 3, light blue bar). (c) A volcano plot illustrating differentially expressed genes in six CYLD cutaneous syndrome tumours and three normal skin samples from material taken at level 1. Genes with a fold change of > 2 and an adjusted *P*‐value of < 0·05 after correction for multiple hypothesis testing are indicated using green dots. *NTRK2* and *NTRK3* expression is indicated. (d) Immunoblotting of frozen sections from level 1 to investigate phosphorylated mitogen‐activated protein kinase (ERK) status, with total ERK expression for normalization. Glyceraldehyde 3‐phosphate dehydrogenase (GAPDH) is used as a loading control. Samples treated with active drug are indicated using a plus sign, and placebo using a minus sign. (e) Immunohistochemical staining of tissue sections of cylindroma from level 1 with B‐cell lymphoma (BCL)2 antibody (#15071, Cell Signaling Technology, Beverly, MA, U.S.A.), counterstained with haematoxylin (original magnification × 20; scale bar = 100 μm). A negative control image performed without the primary antibody is shown in the inset.

We studied 28 skin tumour biopsies from 14 patients (who provided consent) in a clinical trial assessing the utility of targeting tropomyosin receptor kinase in CCS. Ethical approval was obtained for this study (National Research Ethics Service Committee North East–Tyne and Wear Ref:14/NE/080; ISRCTN 75715723).^3^ Briefly, skin tumours in CCS, such as cylindroma and spiradenoma, were treated for 12 weeks with either active treatment (pegcantratinib 0·5% w/w) or matched placebo, prior to skin biopsy (full protocol detailed elsewhere).^4^ We sought to investigate drug concentration, transcriptomics and protein data using diverse methodologies from a single 4–6‐mm diameter punch biopsy taken from the centre of each tumour, which was snap frozen in liquid nitrogen. To carry out this investigation, we optimized a serial sectioning protocol (Fig. 1a) that allowed tumour material to be obtained from different measured levels of the punch biopsy, with confirmation of position using standard histology of adjacent sections. Precise cryosectioning is central to this process, with every section accounted for in order to achieve the measurements indicated. All depths indicated are calculated based on the number of sections taken, and as such are reported as an approximate depth owing to inherent minor variations associated with cryosectioning.

We assessed drug concentrations at three levels within the tumour biopsy using a mass spectrometry‐based assay (liquid chromatography–mass spectrometry/mass spectrometry), giving an indication of drug penetration (Fig. 1a). A representative example taken from two patients is shown in Figure 1b. Tissue sections were also taken adjacent to levels subject to drug measurement (Fig. 1a, c) for RNA extraction. High‐quality RNA (mean RNA integrity number of 9·5) was obtained across the 28 samples.^5^ Differential gene expression of six CCS tumour samples (using RNA extracted from level 1) compared with normal epidermis from three unaffected control patients is indicated in the volcano plot, performed using the DeSeq2^6^ software package (Fig. 1c).^3^ This demonstrated expression of *NTRK2* and *NTRK3* genes, which are known to encode the protein targets of pegcantratinib. Histology sections (level 1) were also obtained to assess expression of proteins regulated by TRK signalling, such as mitogen‐activated protein kinase (ERK) and B‐cell lymphoma (BCL)2. Phosphorylated and total ERK status (Fig. 1d), and immunohistochemical assessment of BCL2 (Fig. 1e) were obtained as previously described.^7^ We successfully obtained drug concentration data (28 of 28 tumours analysed), RNAseq data (24 of 24 tumours analysed), BCL2 expression (28 of 28 tumours analysed) and pERK status (26 of 28 tumours analysed).

Serial sectioning has previously been used to determine drug penetration in the skin,^8^ but this has not been coupled with transcriptomics or protein expression data. The method described here offers the ability to correlate data from a variety of molecular assays from adjacent sections of a single piece of human biopsy material; other assays including genome sequencing, proteomics and metabolomics may also be feasible.

Caveats to our method apply. The thickness of the diseased skin that was studied may limit the application of this method; the total depth of the biopsy required in this study was approximately 1·5 mm. Modifications to the number of levels obtained will allow for the study of superficial skin diseases, and optimization can be guided by the histological sections obtained. The extent of gene expression changes will vary with the drug type and penetration in different skin diseases. In addition, we demonstrate data from diverse assays from adjacent sections, not the same cells, owing to technical limitations relating to current assay technology. Nevertheless, our proof‐of‐principle work in skin tumours in CCS provides a novel method that could be adapted to study other topically treated skin tumours or diseases.
